# Effects of *Foeniculum vulgare *ethanol extract on osteogenesis in human mecenchymal stem cells

**Published:** 2013

**Authors:** Zahra Mahmoudi, Masoud Soleimani, Abbas saidi, Gholamreza Khamisipour, Arezoo Azizsoltani

**Affiliations:** 1 *Department of Biotechnology, Faculty of New Technologies and Energy Engineering, Shahid Beheshti University, Tehran, I. R. Iran*; 2 *Department of Hematology,Faculty of Medical Sciences, Tarbiat Modares University, Tehran, I. R. Iran*; 3 *Department of Hematology, School of Medicine, Boushehr University of Medical Science, Boushehr, I. R. Iran*; 4 *Department of Biotechnology, Faculty of Agriculture, Bu-ali Sina University, Hamedan, I. R. Iran*

**Keywords:** Alkaline Phosphatase, *Foeniculum Vulgare*, Osteogenesis, Proliferation

## Abstract

**Objective:** Osteoporosis or silent disease is a major bone disorder in elderly women in current century. Estrogen has an important role in osteogenesis and prevention of bone fractures. Hormone replacement therapy (HRT) is usually accompanied by such effects as breast and ovary cancers. Thus, there is an increasing demand for replacement with plant phytoestrogens. This study is focused on determining the effects of *Foeniculum vulgare* extract on proliferation and osteogenesis progress in human mesenchymal stem cells.

**Materials and Methods**: Human mesenchymal stem cells were isolated and treated with different amount of plant extracts (0.5 to 100 µg/ml). Extract cytotoxicity was measured using MTT assay. The alkaline phosphatase enzyme activity was measured to evaluate the differentiation progress.

**Results: **Results of MTT assay and alkaline phosphatase activity showed that Foeniculum vulgare extract, at range of 5 to 50 µg/ml, may positively affect cell proliferation and mineralization. The most proliferation and enzyme activity were seen with dose of 5 µg/ml.

**Conclusions:** *Foeniculum vulgare* has been used in Iranian folk medicine for many years. Our in vitro study showed that *Foeniculum vulgare* extract has osteoprotective effects.

## Introduction

Osteoporosis, one of the most prevalent diseases in current century, is defined as microarchitecture deterioration of bone by reducing bone density (Gronholz, 2008 ▶). Osteoporosis is mostly caused by an imbalance between the function of osteoblast and osteoclast cells leading to bone resorption in bone remodeling process (Pagani et al., 2005). Bone remodeling is a restructuring process of existing bones, which is in constant resorption and formation (Meunier, Delmas et al., 1999 ▶). When this process becomes unbalanced, bone pathology appears (Kushida Takahadhi et al., 1995 ▶). Estrogen limits bone loss through its effects on osteoblast and osteoclast activity (Christian, Christian et al., 1982 ▶). Estrogen activation of osteoblasts stimulates expression of the special proteins and growth factors (Tielens, Wymeersch et al., 2008 ▶). Reduction in estrogen leads to increased osteoclastic activity because of reduced hormonal control over osteoblast cells activity (Huang, Ettinger et al., 2007). During the past decades, HRT or Hormone Replacement Therapy has been used as the major treatment for osteoporosis (New, Robins et al., 2000 ▶). This method is effective in preventing bone loss and reduces the risk of fractures in postmenopausal women. In recent years, there are concerns about using this method due to the risk of developing breast and endometrial cancers and unwanted side effects associated with these chemical steroids (     Neuner, Zimmer et al., 2003 ▶) . As a result, scientific research has focused on finding new natural replacements for chemical drugs. Plants can provide potential alternative as antiosteoporotic agents because they constitute a rich source of bioactive secondary metabolites. Moreover, they can degrade to non-toxic products (Horiuchi, Onouchi et al., 2000 ▶). Phytoestrogens are plant derived non- estradiol phenolic compounds which are believed to protect against cardiovascular diseases, osteoporosis, and hormone-related disorders (Horiuchi, Onouchi et al., 2000 ▶). Phytoestrogens can be easily metabolized and eliminated. Many investigations have shown lower prevalence of osteoporosis and hip fracture among Asian women consuming high amount of phytoestrogens (Knight and Eden, 1996 ▶; Hsu, Hsu et al., 1999 ▶).

Fennel (*Foeniculum vulgare* Mill) is an umbilliferous plant. The fruit and root infusions are used as relaxant, estrogenic, analgesic, and anti-inflammation agent. (Ozbek et al., 2003). Fennel is used in herbal remedies for respiratory tract disorders and indigestion and is also used to increase milk flow in nursing mothers (Choi et al., 2004 ▶;      Amjad and Jafary 2000  ▶). Fennel seeds have been shown to increase milk secretion, promote menstruation, facilitate birth, and alleviate the symptoms of dysmenorrhea (   ).  

Fennel seeds extract increases libido and female climacteric (Ostad et al., 2001 ▶; Namavar Jahromi et al., 2003 ▶). A study conducted by Mimica Dukic et al., 2003, showed an antifungal activity of Fennel essential oil. Moreover, different doses of Fennel essential oil (25 and 50 µg/ml) significantly decreased level of oxytocin and prostaglandin E and induced uterine contractions in primary dysmenorrhea (Ostad et al., 2001 ▶; Namavar Jahromi et al., 2003 ▶). Fennel seed extract has been shown to have estrogenic, antioxidant, and antihirsutism activities (Oktay et al., 2003 ▶; Malini et al., 1985; Javadnia et al., 2003).

Therefore, based on its estrogenic activity, the present study was carried out in an attempt to evaluate the potential activity of ethanolic extract of Fennel roots in proliferation and alkaline phosphatase enzyme activity in human mesenchymal stem cells. All procedures of current study were performed at the Department of Hematology of Tarbiat Modares University and Hematology Research Center of Shariati Hospital of Tehran, Iran.

## Materials and Methods

**Chemicals and reagents**

Dulbecco's Modified Eagle's Medium (DMEM) and fetal bovine serum (FBS), pen/strep were purchased from (Gibco, BRL, Grand Island, NY, USA) and L-ascorbic acid and β-glycerophosphate, dexamethasone, 17β-Estradiol (E2) and DMSO were purchased from (Sigma-Aldrich, MO, USA). 

**Cell extraction and culture**

Human bone marrow mesenchymal stem cells were extracted in Hematology Research Center of Shariati Hospital of Tehran. Briefly, bone marrow which aspirated from iliac crest of healthy female donors after formal consent was collected with added heparin (6000 U). Mononuclear cells were isolated by centrifugation at 1100 *g* for 30 min. The cells were rinsed twice with PBS and maintained in complete medium (low glucose DMEM with 10% fetal bovine serum; 2 mM _L_-glutamine, 100 U/ml penicillin and streptomycin) at 37 °C in humid 5% CO_2_ air atmosphere. 

**Preparation of plant extract**

Dried and powdered roots of Fennel were soaked in 95% ethanol for 48 h and FE was obtained by extraction with 70% ethanol (in water, v/v) at room temperature for several times. The extract was filtered and concentrated with a rotary evaporator. The extract was then dissolved in dimethylsulfoxide (DMSO) to a final concentration of 20 mg/ml and diluted in culture medium to the working solution before use. The extract was filtered and stored at 4 °C.

**Cell viability and proliferation assay**

hMSCs were cultured in 96-well flat-bottom plate in density of 5×10^3 ^per well. After 24 h of incubation, cells were exposed to (0.5, 1, 5, 10, 50, and 100 µg/ml) Fennel extract. Cells then were incubated at 37 ºC for 1, 2, or 3 days. Finally, 20 µl of MTT (5 µg/ml) was added and the samples were left to incubate for 4 h. The medium was discarded and the formazan salts were dissolved in DMSO (100 µl) at 37 °C for 30 min. The end product color was then analyzed by measuring absorbance at 540 nm with a reference wavelength at 630 nm. The data are expressed as percent of cell viability compared with that of control culture, defined as 100%.

**Osteogenic differentiation and treatment with fennel extract**

hMSCs were seeded on to 12-well plates at a density of 1×10^5 ^Per well. When over 80% confluency was reached, osteogenesis was induced by the α-MEM medium with osteogenic supplement, containing DMED+10% FBS supplemented with 0.2 mM L-ascorbic acid-2-phosphate, 10nM dexamethasone, 10mM β-glycerophosphate with different concentration of Fennel extract. The medium was replaced every 3 days. On days 7 and 14, cells were collected for measuring ALP activity. Triplicate tests were conducted in each experiment. 

**Measurement of alkaline phosphates (ALP) activity**

hMSCs were cultured in 35 mm culture dishes for 24 h as described above and then treated with Fennel extract (0.5, 1, 5, and 10 µg/ml) for 15 days. Synthetic estrogen 17β-estradiol (Sigma-aldrich, MO, USA) in final concentration of 10^-8 ^M as control group. To determine the level of alkaline phosphatase activity, total cell protein was extracted using 200 µl RIPA buffer. The lysate was then centrifuged at 14,000×g at 4 ºC for 15 min. Supernatant was collected and ALP activity was measured with ALP assay kit (Parsazmun, Tehran, Iran) using *p-nitrophenyl phosphate* (*p-NPP)* as substrate and alkaline phosphatase provided in the kit as standard. The activity of enzyme (IU) was normalized against total protein content on cell lysate. In this study, ALP activity is expressed as nmol (P-nitrophenyl)/mg protein.

**Statistical analysis**

Data are expressed as mean±standard deviation (SD). Statistical significances were analyzed using the ANOVA test. p<0.05 was considered significant. 

## Results

**Figure1:**
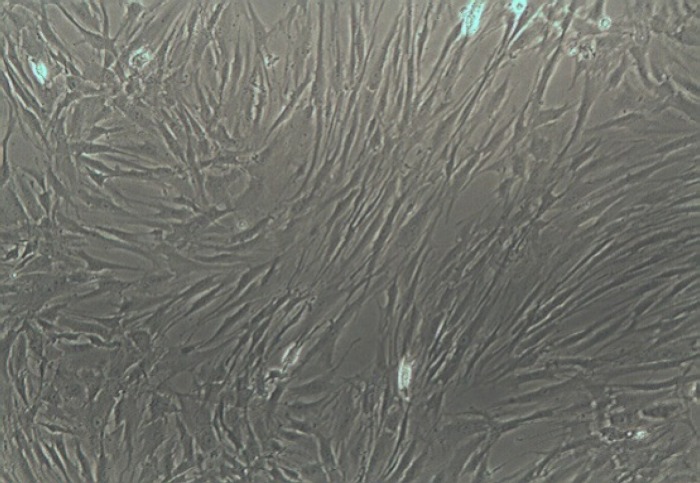
Morphology of human mesenchymal bone marrow stem cells before inducing with osteogenesis media

**Figure2:**
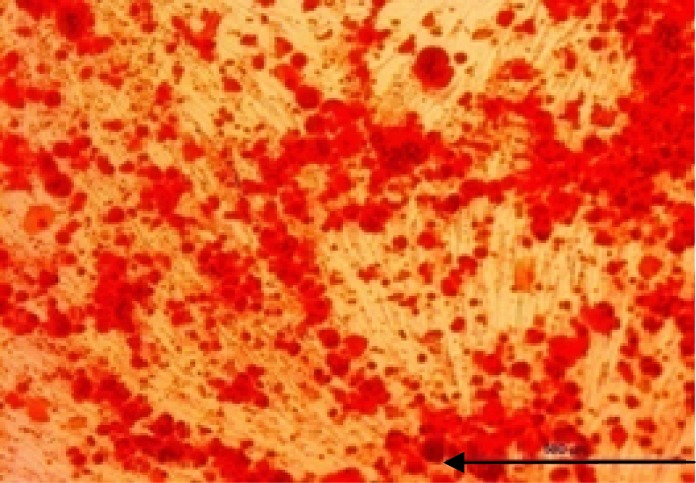
Mesenchymal bone marrow stem cells after inducing with osteogenesis media. The Alizarin red staining was performed in day 21 of osteogenesis progress. Calcium nodule formation confirmed differentiation ability of the cells.

**Figure 3:**
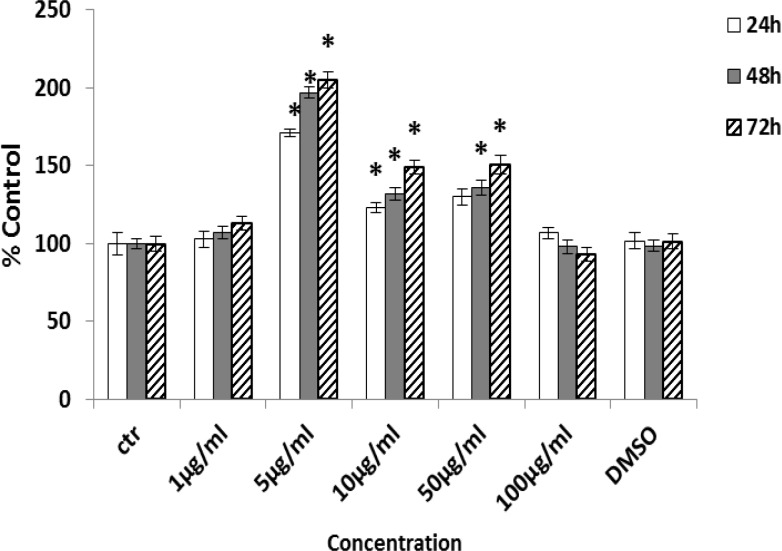
The extract dose-response increased the proliferation of hMSCs. hMSCs were cultured with medium and various concentrations extract (0.5 to 100 µg/ml) for three days. Each point represents the mean±SD. of four determinations. * p<0.05, compared with the control (Medium).

Processing of human mesenchymal stem cells isolation was accomplished as previously described. The morphology of human Mesenchymal Stem Cells is shown in (Figure1). The phenotype of hMSCs was evaluated by flow cytometry analysis. After 4 cell passage, the surface positive markers CD44, CD73 and CD105 of MSCs were assayed. Cells were incubated in a humidified incubator equilibrated with 5% CO2 at 37°C. For further approval, differentiation to osteoblast and Alizarin red staining were performed (Figure 2). Effects of Fennel Extract on hMSCs viability is shown in (Figure 3). The effects of extract were examined at concentration range from 1 to 100 µg/ml, in times interval of 24, 48, and 72 h. Although, there was a decrease in cell viability at 100µg/ml, none of the applied concentrations were toxic for hMSCs. Significant statistical differences were observed among the treated samples proliferation activity and that of the control (p<0.05). 

**Figure 4:**
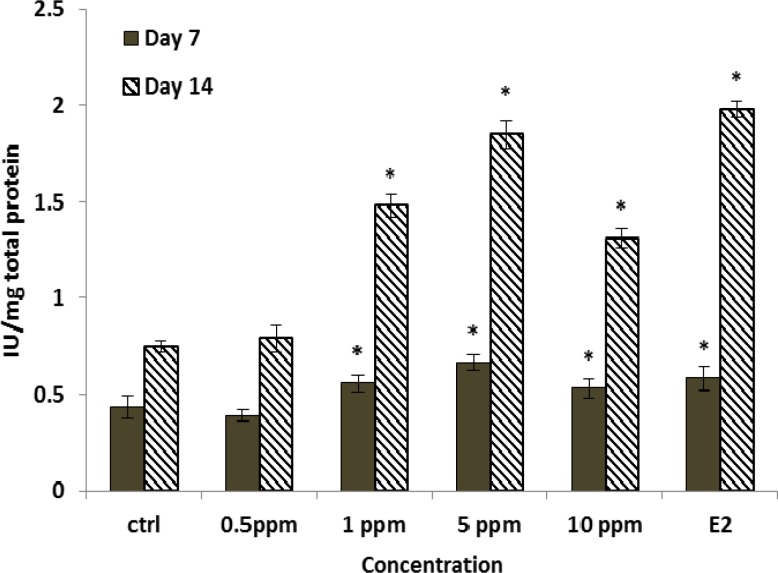
Effects of extract on ALP activities of hMSC cells. Cells were cultured in medium, ethanol extract (0.5 to 10 µg/ml), and E2 (10^-8 ^M) for 15 days in presence of osteogenesis media, Results are expressed as means±SD (* p<0.05), compared with the control (DMEM+10% FBS).

The maximum cell proliferation was observed in concentration of 5 µg/ml at 48 h after treatment. Consequently, we could come to conclusion that the extract could stimulate proliferation of human mesenchymal stem cells.hMSCs were cultured with vehicle or various concentration of Fennel extract. E2 and ALP activity, as a marker of osteoblast differentiation, was measured during induction of osteogenesis. Results indicated that Fennel extract significantly increased the ALP activity of hMSCs compared with the control in a dose-dependent manner (Figure 4) and higher activity was observed on day 14 at concentration 1 μg/ml (p<0.05) which was comparable with E2 group (Figure 4).

## Discussion

To investigate the effects of *Foeniculum vulgare* ethanol extract on osteogenesis, well-recognized human bone marrow mesenchymal stem cells were selected as *in vitro *model. In this study, we examined cell growth and proliferation in hMSCs in vitro. Result showed that low concentrations of ethanol extract could stimulate cell proliferation and alkaline phosphatase enzyme activity. Proliferation of hMSCs was significantly increased in range of 5-50 µg/ml and the most proliferation was seen at 5 µg/ml of extract. Effects of plant extracts on cell proliferation have been examined in many prior investigations. For example, effects of Green Tea catechin were examined on MC3T3-E1 cell line in range of (1-100 µg/ml) and the most proliferation was observed in 1 µg/ml concentration. These result are in line with the result of the current study (Wei et al., 2011 ▶).

The integrity of the skeleton requires a dynamic balance between bone formation and bone resorption, which are controlled by hormones and cytokines (Kushida et al., 1995 ▶). Estrogen plays an important role in physiological regeneration of bones and decreasing level of serum estrogen is associated with osteoporosis after menopause in elderly women (Tielnse et al., 2008 ▶). There are many studies to prove the effect of estrogen as antiosteoporotic agent. The effects of estrogen on osteoblasts and osteoclasts are mediated by binding to intracellular estrogen receptor and modulating the production of target proteins (Jung et al., 2010 ▶). Phytoestrogens, with similar structure to 17β-estradiol (E_2_), are known to produce a protective benefit on osteoporosis in post-menopausal women. These compounds are suggested to have not only estrogenic activities such as stimulation of uterus growth and inhibition of bone loss, but also anti-estrogenic activities such as inhibition of breast cancer cell growth (News et al., 2000 ▶). Fennel extract are a rich source of phytochemicals and many of these compounds have beneficial effects on human health. The similarity in action between phytoestrogen and estradiol promoted us to investigate whether Fennel extract phytochemicals might exhibit estrogenic activity in cell culture system. Stem cells can promote bone healing through extensive proliferation, differentiation, and growth factor secretion in the local microenvironment at the site of injury. In this study, we demonstrated that Fennel extract (5 -50 µg/ml) can stimulate proliferation of human MSCs in a dose- and time-dependent manner. Reaching peak at 72 h and showing inhibitory effects at high concentration (100µg/ml). The proliferation pattern of cells exposed to Fennel extract was biphasic. The Fennel extract at low concentrations stimulated proliferation of MSCs and became cytotoxic at relatively high levels. Similar biphasic effect has been noted for other phytoestrogens such as genistein in soy bean, glabridin, and glabrene isolated from licorice root (Zava et al., 1997; Somjen et al., 2004; Choi, 2005 ▶).

In the present study, effects of Fennel extract on ALP activity in human bone marrow mesenchymal stem cells were examined. ALP activity is one of the osteoblastic markers and has substantial function in mineralization. Our results indicated that the Fennel extract significantly induced the ALP activity in range of (1-10 µg/ml) in contrast with the control group and the most enzyme activity was observed at 5 µg/ml. Most ALP activity was observed with 1 nM and the inducing effects of extract decreased with increasing the extract concentrations. This observation is in agreement with a study which carried out on effect of *Ulmus davidiana* plant extract. The study was evaluated on MC3T3_E1 preosteoblastic cell line in range of (1-50 µg/ml). The most ALP activity was observed with 1 µg/ml (Suh et al., 2007 ▶).

ALP activity significantly increased in presence of Fennel extract. ALP is known to be critically involved in initiation of mineralization during bone formation. Its activity, along with other specialized bone proteins, renders extracellular matrices suitable for mineral deposition (Perets et al., 1996 ▶). Up-regulation of ALP activities was noted after 4–14 days of Fennel extract treatments with maximum activity on day 14. 

Many studies have been carried out on Foeniculum vulgare essential oil (Bilia et al., 2002; Yamini et al., 2002). More than 80% of the essential oil components of Fennel is composed of *trans*-Anatole.            (Singh, Maurya et al., 2006 ▶) . As mentioned previously, *trans*-Anatole appears to have an estrogenic activity. It seems that an important part of antiosteoporotic effects of Fennel extract may be due to the presence of *trans*-Anatole, although further works are needed to clarify the role of other components of Fennel extract in osteogenesis (           Nakagawa and Suzuki 2003  ▶;      Tognolini, Ballabeni et al., 2007 ▶). 

By applying different concentrations of Fennel extract, this study showed that Fennel extract can enhance human BMSCs proliferation and their differentiation toward the osteoblast lineage. Further investigations regarding its beneficial effects and other aspect relevant for extrapolation to human exposure seem necessary.
